# Hormonal aspects of overtraining syndrome: a systematic review

**DOI:** 10.1186/s13102-017-0079-8

**Published:** 2017-08-02

**Authors:** Flavio A. Cadegiani, Claudio E. Kater

**Affiliations:** From the Adrenal and Hypertension Unit, Division of Endocrinology and Metabolism, Department of Medicine, Escola Paulista de Medicina, Universidade Federal de São Paulo (EPM/UNIFESP), R. Pedro de Toledo 781 – 13th floor, São Paulo, SP 04039-032 Brazil

**Keywords:** Overtraining, Overreaching, Adrenal, Cortisol, Hormone, Sports endocrinology

## Abstract

**Background:**

Overtraining syndrome (OTS), functional (FOR) and non-functional overreaching (NFOR) are conditions diagnosed in athletes with decreased performance and fatigue, triggered by metabolic, immune, hormonal and other dysfunctions and resulted from an imbalance between training stress and proper recovery. Despite previous descriptions, there is a lack of a review that discloses all hormonal findings in OTS/FOR/NFOR. The aim of this systematic review is to evaluate whether and which roles hormones play in OTS/FOR/NFOR.

**Methods:**

A systematic search up to June 15^th^, 2017 was performed in the PUBMED, MEDLINE and Cochrane databases following PRISMA protocol, with the expressions: (1)overtraining, (2)overreaching, (3)overtrained, (4)overreached, or (5)underperformance, and (plus) (a)hormone, (b)hormonal, (c)endocrine, (d)adrenal, (e)cortisol, (f)GH, (g)ACTH, (h)testosterone, (i)IGF-1, (j)TSH, (k)T4, (l)T3, (m)LH, (n)FSH, (o)prolactin, (p) IGFBP-3 and related articles.

**Results:**

A total of 38 studies were selected. Basal levels of hormones were mostly normal in athletes with OTS/FOR/NFOR compared with healthy athletes. Distinctly, stimulation tests, mainly performed in maximal exercise conditions, showed blunted GH and ACTH responses in OTS/FOR/NFOR athletes, whereas cortisol and plasma catecholamines showed conflicting findings and the other hormones responded normally.

**Conclusion:**

Basal hormone levels are not good predictor but blunted ACTH and GH responses to stimulation tests may be good predictors of OTS/FOR/NFOR.

## Background

Overtraining syndrome (OTS) and related states are results of a combination between excessive overload in training stress and inadequate recovery, which leads to acute feelings of fatigue and decreases in performance [1]. The whole spectrum of underperformance conditions includes: a. functional overreaching (FOR), when there is a very short-term (days to few weeks) decrement in performance and *supercompensation* (improvement in performance) after recovery [1]; b. non-functional overreaching (NFOR), when performance worsens for a short period (but longer than FOR, between weeks to months) and a full recovery (although not always the previous performance capacity is reestablished) is observed after proper recovery period [1]; and c. overtraining syndrome (OTS), when a long-term (usually several months but can be indefinitely) decrement in performance capacity allied to psychological symptoms are seen [1]. Despite of these descriptions, it is unfeasible to set an unquestionable definition and precise limits between OTS, NFOR and FOR.

The imbalance between training and recovery, which can be worsened or confounded by inadequate nutrition, illness, psychosocial stressors and sleep disorders [1], among many other causes, leads to dysfunction of pathways and responses in immune, inflammatory, neurological, hormonal and metabolic systems as a maladaptation to chronic exposure to extreme metabolic and tissue environments. This includes, for instance, chronic glycogen depletion (although normal levels of glycogen are found at the moment of the examination of athletes).

There are apparently no reliable or accurate biomarkers that help diagnose OTS/NFOR/FOR, even though diminished maximal lactate concentration, creatine kinase altered reaction to eccentric and new-onset exercises and decreased plasma glutamine levels have been found [1]. Despite of the efforts to find tools to improve diagnosis of OTS/NFOR/FOR, these have not yet been successful. Regarding the current literature on endocrine aspects of OTS/NFOR/FOR, basal (resting) hormone measurements cannot distinguish between athletes who successfully adapt to FOR and those who fail to adapt and develop symptoms of OTS, although a diminished hormone reserve and consequent impairment of hormone response to acute stressful situations may be one of the triggers of OTS/NFOR/FOR symptomatology, particularly the worsened performance, the key symptom of any underperformance syndrome*.* Regarding hormonal aspects of OTS/NFOR/FOR, the latest guideline [1] on OTS/NFOR/FOR recommends that further studies are recommended to discover possible hormonal diagnostic tests [1].

Therefore, to determine if and which basal or stimulated hormonal markers can be successfully linked to OTS/NFOR/FOR and which are the most accurate predictors is the aim of this systematic review. The primary outcomes are an evaluation of methodology for the assessment of overtraining, a comparison of hormonal tests performed and analysis of results of studies involving correlation between hormones and OTS/NFOR/FOR.

## Methods

### Search strategies

To provide a wide, complete and thorough systematic review over hormonal aspects in OTS/NFOR/FOR, PRISMA protocol for systematic reviews was used for the study design and a systematic search was conducted through the electronic PUBMED, MEDLINE (Ebsco), and COCHRANE databases, from their earliest records to November 8th, 2017. Searched expressions were: (1)overtraining, (2)overreaching, (3)overtrained, (4)overreached or (5)underperformance, and (plus) (a) hormone, (b) hormonal, (c) endocrine, (d) adrenal, (e) cortisol, (f) GH, (g) ACTH, (h) testosterone, (i) IGF-1, (j) TSH, (k) T4, (l) T3, (m) LH, (n) FSH, (o) prolactin and (p) IGFBP-3, in a total of 85 expressions searched.

We also analyzed articles that were mentioned within identified studies whenever OTS/NFOR/FOR or a similar disorder was described (e.g. cortisol and adrenocorticotropic hormone (ACTH) profiles and over-trained athletes).

### Data extraction

All studies were evaluated after removal of duplicate articles, according to: 1) authorship, 2) publication date, 3) studied population, 4) definition of overtraining and overreaching syndromes, 6) study design and methods, 7) methods of hormone assessment, 8) results, 9), conclusions and 10) study variables and bias.

### Eligibility criteria

Inclusion criteria were: 1) whole article written in English; 2) hormone profile (basal, stimulated, or both) and correlation to OTS/NFOR/FOR states as an outcome (but not necessarily the primary outcome); 3) absence of influence of any hormone therapy; 4) absence of confounding diseases that would lead to an impaired hormonal status caused by the disorder itself, which would also exclude the diagnoses of OTS/NFOR/FOR; 5) population with proper diagnosis of OTS/NFOR/FOR; and 6) studies with humans.

### Quality assessment

The abstract of each of researched study was analyzed by one of the authors (F.A.C.) and assessed for inclusion according to the eligibility criteria. The studies that fulfilled the inclusion criteria were evaluated with regard to their rationale, study design, primary outcomes, assessment of OTS/NFOR/FOR diagnosis, statistical analysis, results, discussion and conclusions. Studies that presented any bias in methodology, results, or interpretation of the exposed data, that could interfere in the analysis were excluded. Studies whose methodology could not be assessed by the abstract had the full text analyzed, prior to the decision of the acceptance for the systematic review.

### Statistical analysis

Due to the heterogeneity of the studies that aim to correlate hormone responses and OTS/NFOR/FOR, a meta-analysis, our initial idea, was unfeasible. Moreover, since several variables were taken into account, including the type of sports performed, type of tests and stimulus performed, and whether hormones were analyzed basally, and during a resting period or resting after an intensive training load, analysis of each aspect was performed separately, and in several different combinations, including studies that performed equivalent methods, although not similar, including all tests stimulated by exercises, regardless of the type of stimulation, tests performed in endurance sports, regardless of the type of sport, and resting combined with basal hormones, regardless of the previous exercise stimulation. Data was also analyzed according to the number of studies investigating each type of sport, the number of athletes with OTS/NFOR/FOR and healthy athletes, and the number of studies using each type of hormone stimulation test were quantified. Study results were analyzed in terms of percentage of type of responses for each of the tests performed. Once meta-analysis was not performed, *t*-tests, one-way ANOVA and non-parametric tests were not employed, and therefore a statistical analysis program was not necessary.

## Results

### Selection of the studies

The initial search yielded 835 articles. The sum of the searches for “overtraining”, “overreaching”, “overtrained”, “overreached” and “underperformance”: 1. with “catecholamines” yielded 62 articles; 2. With “ACTH” yielded 29 articles; 3. with “cortisol” yielded 161 articles; 4. with “adrenal” yielded 60 articles; 5. “GH” yielded 13 results; 6. with “IGF-1” yielded 19 articles; 7. with “IGFBP-3” yielded four articles; 8. with “testosterone” yielded 100 articles; 9. with “LH” yielded nine articles; 10. with “FSH” yielded four articles; 11. with “TSH” yielded four articles; 12. with “T4” yielded 12 articles; 13. with “T3” yielded 13 articles; 14. with “prolactin” yielded 10 articles; 15.with “hormone” yielded 195 articles; 16. with “hormonal” yielded 92 articles; and 17. with “endocrine” yielded 49 articles.

However, 612 studies were yielded in more than one search, while 185 did not meet criteria to be included in this systematic review. None of the studies were in a language other than English. Ultimately 38 articles were included for analysis in this systematic review [2–38] (detailed in Table 1), representing 4.59% of the original search results. Two studies [34, 35] were included in the same analysis as both analyzed different hormones in the same subjects, in both moments.

**Table 1:** Description of selected studies

Authorship, year of publication and reference	Number of subjects	Sport(s)	Type of athletes and type of analysis	Basal levels	Functional tests and responses
Aissa et al., 1999 [25]	36	Volleyball, soccer and karate	AA (CC)	↓IGFBP-3/nl IGF-1/nl IGFBP-1/↑IGF-1/IGFBP-3 ratio	-
Aubry et al., 2015 [2]	31	Triathlon	AA (RRH)	-	OTP (↓serum catecholamines)
Barron et al., 1985 [38]	6	Long distance running	AA (CC, CRH)	↑cortisol/↑ACTH	ITT (↓cortisol/↓ACTH)
Booth CK et al., 2006 [16]	43	Multiple (military activities)	PHA (CHB)	-	OTP (↓T/C/nl free T/nl cortisol)
Coutts et al., 2007 [13]	18	Rugby	PHA (CHB, RRH)	-	OTP (Nl total T/↓Total T/↓T/C/nl ACTH/nl cortisol/nl insulin/nl nocturnal urinary catecholamines)
Coutts et al., 2007 [14]	7	Rugby	PHA (CHB)	-	OTP (↓T/C/nl total T/nl cortisol)
Coutts et al., 2007 [15]	16	Rugby	PHA (RRH)	-	OTP (Nl T// nl total T/nl cortisol)
Elloumi et al., 2005 [18]	11	Rugby	AA (CC, CRH, RRH)	Nl IGF-1/nl IGFBP-3	ME after OTP (↓IGFBP-3/↑IGF-1/nl IGF-1/↑IGF-1/IGBP-3 ratio)
Fry et al., 2006 [17]	16	Weight-lifting	PHA (CHB, RRH)	-	OTP (↑Nocturnal urinary Catecholamines/↓muscle beta2 receptors)
Fry et al., 1998 [27]	17	Weight lifting	AA (CC, RRH)	Nl GH/nl Pep F/↓TotalT/nl freeT,	OTP (Nl GH/Nl Pep F/↓TotalT/↓freeT/↓T/C/↓FreelT/C)
Fry et al., 1994 [31]	17	Weight-lifting	AA (CC, CRH)	Nl Plasma Catecholamines	ME (↑Plasma Catecholamines)
Fry et al., 1992 [34]	5	Middle distance running	PHA (CHB)	-	OTP (Nl total T/nl cortisol/nl SHBG/nl LH/nl FSH/nl T/C)
Hedelin et al., 2000 [23]	9	Rowing	PHA (CHB)	-	CSP (↓ cortisol/nl total T)
Hoogeveen et al., 1996 [30]	10	Cycling	PHA (CHB, CB)	-	a. Resting levels after OTP (Nl total T/nl LH/nl cortisol) b. ME after OTP (Nl total T/nl LH/nl cortisol)
Hooper et al., 1993 [32]	14	Swimming	AA (CC)	↑Norepinephrine	-
Hug et al., 2003 [22]	11	Cycling	PHA (CHB)	-	OTP (↑Total T/↑free T/↓cortisol/nl SHBG/↓GH/nl IGF-1/nl IGFBP-3/nl T/C)
Hynynem et al., 2006	24	Multiple (military activities)	AA (CC)	Nl nocturnal catecholamines/nl nocturnal cortisol	-
Kraemer et al., 2004 [21]	25	Soccer	PHA (CHB, RRH)	-	CSP (Nl Total T/nl cortisol)
Lehmann et al., 1992 [35]	8	Middle distance running	PHA (CHB, CB)	-	a. Resting levels after OTP (↓cortisol/↓aldosterone, nl total T/nl insulin/nl pep C, nl GH/nl prolactin/nl FSH/nl LH/nl TSH/nl T3/nl T4, ↓nocturnal urinary catecholamines/nl plasma catecholamines) b. ME after OTP (↓cortisol/↓aldosterone/nl testosterone/insuli/nl pepC/nl GH/nl prolactina/nl FSH/nl LH/nl TSH/nl plasma catecholamines)
Lehmann et al., 1990 [36]	9	Middle distance run	PHA (CHB, CB)	-	a. Resting levels after OTP (Nl cortisol/↑aldosterone/nl total T/nl insulin/nl pep C/nl GH/nl prolactin/nl FSH/nl LH/nl TSH/nl T3/nl T4/↓NUC/nl plasma catecholamines/nl 24 h UFC) b. ME after OTP (Nl cortisol/↑aldosterone/nl testosterone/nl insulin/nl pepC/nl GH/↓prolactin, nl/FSH/nl LH/↓TSH/↓ plasma catecholamines)
Le Meur et al., 2014 [3]	35	Triathlon	PHA (CHB, RRH)	-	OTP + CSP (↓serum catecholamines)
Le Meur et al., 2013 [4]	35	Running	PHA (CHB, RRH)	-	OTP (Nl serum catecholamines)
Mackinnon et al., 1997 [29]	24	Swimming	AA (CC)	↓NUC Nl plasma cathecolamines/nl T/C/nl total T	-
Meeusen et al., 2010 [9]	8	Long distance run/Triathlon/Cycling/Motor Cross/Short distance run	AA (CC, CRH)	↑GH/↑ACTH	TBE (OTS compared to OR) (↓GH/↓ACTH)
Meeusen et al., 2004 [20]	7	Cycling	AA (CC, CRH)	Nl cortisol, prolactin/nl GH/nl ACTH	TBE (OTS compared to control) (↓GH/↓ACTH/↓cortisol/↓prolactin)
Moore et al., 2007 [12]	9	American football	PHA (CHB)	-	OTP (↓Total T/nl cortisol/nl T/C)
Nederhof et al., 2008 [11]	3	Skaters	AA (CC, CRH)	↓cortisol/nl ACTH	TBE (nl ACTH/nl cortisol)
O’Connor et al., 1989 [37]	14 women (3 NFOR	Swmming	AA (CC)	↑16 h salivary cortisol	-
Rietjens GJ et al., 2005 [19]	7	Not specified	PHA (CRH, CHB, RRH, CB)	Nl GH/nl IGF-1/nl ACTH/nl cortisol	a. Resting levels after OTP (Nl GH/nl IGF-1/nl ACTH/nl cortisol) b. Short ITT after OTP (Nl GH/nl IGF-1/nl ACTH/nl cortisol)
Roberts et al., 1993 [33]	5	?	PHA (CHB)	-	OTP (↓ total T/↑cortisol/↓T/C/↓fertility)
Schmikli et al., 2012 [5]	21	Soccer	AA (CC, CRH)	↓GH/nl ACTH/nl cortisol	ME (↓GH/↓ACTH/nl cortisol)
Schmikli et al., 2011 [7]	15	Soccer/Middle-long distance run	AA (CC, CRH)	nl cortisol/nl ACTH	ME (↓cortisol/decoupled ACTH to cortisol response/nl ACTH)
Slivka et al., 2010 [10]	8	Cycling, Triathlon	PHA (CHB, CB)	-	a. ME after OTP (↓salivary cortisol/↓salivary testosterone/↑T/C) b. Resting levels after OTP (↓salivary cortisol/nl salivary testosterone)
Steinacker et al., 2000 [24]	10	Rowing	PHA (CHB)	-	OTP (Nl total T, nl cortisol, nl DHEA-S/↓LH/↓FSH/nl aldosterone/↓insulin/↓pep C/nl prolactin/↑GH)
Tanskanen et al., 2011 [6]	Not Specified	Multiple (military)	AA (CC, CRH)	nl T/C ratio/↑cortisol/↑SHBG	ME (↓cortisol)
Thiel et al., 2011 [8]	3	Tennis	PHA (CHB)	-	OTP (↓IGF-1/nl T/C)
Urhausen et al., 1998 [26]	17	Cycling, triathlon	AA (CC, RRH)	Nl Nocturnal Cathecolamines/nl GH/nl cortisol/nl insulin/nl LH/nl FSH/testosterone/nl SHBG/nl T/SHBG/nl T/C/↑ACTH	ME (↓GH/↓ACTH/↓insulin/nl cortisol)
Uusitalo et al., 1998 [28]	15	Middle-distance running, skiing, triathlon and orienting.	PHA (CHB, RRH, CRH)	-	a. Resting levels after OTP (Nl cortisol/nl serum catecholamines/nl testosterone) b. ME after OTP (↓serum adrenaline/nl serum noradrenaline/↓cortisol)

General: *N* number of subjects, *Nl* normal levels, *NS* Not Specified

Parameters: *Pep F* Peptide F (preproencephaline), *TotalT* Total testosterone, *freeT* free testosterone, *T/C* Total Testosterone/Cortisol ratio; *freeT/C* Free Testosterone/Cortisol ratio

Performed tests: *ME* Maximal Exercises, *TBE* Two-Bout Protocol Exercise, *ITT* insulin tolerance test, *OTP* Overload Training Program, *CSP* Competition Season Period

Studies athletes: *PHA* Previously Healthy Athletes, *AA* Athletes affected by OTS/NFOR/FOR

Type of analysis: CC = Basal levels compared with healthy athletes; CHB = Resting hormone levels in FOR-induced athletes compared to previous basal levels of the same subjects when previously healthy; CRH = Acute hormone response to stimulation tests compared to acute response in healthy athletes; RRH = Resting hormone levels to OTP in athletes previously affected by OTS/NFOR/FOR compared to basal hormones in healthy athletes; CB = Acute hormone responses compared to basal levels in the same affected athletes

Among the yielded articles, the reasons of the exclusions were due to: (1) Review of FOR/NFOR/OTS – 57 articles; (2) Performed in animals – 35 articles; (3) Nutritional supplementation for FOR/NFOR/OTS – 20 articles; (4) Hormonal physiological adaption to exercises – 13 articles; (5) Other markers of FOR/NFOR/OTS than not hormones – 14 studies; (6) Markers of stress during training – 09 articles; (7) Other dysfunctions than FOR/NFOR/OTS evaluated (depression, amenorrhea) – 06 studies; (8) Nutritional management of FOR/NFOR/OTS – 06 studies; (9) Improper criteria for diagnosis of FOR/NFOR/OTS – 05 articles; (10) Stimulation therapies effects on FOR/NFOR/OTS (electrical, cryotherapies) – 04 articles; (11) Post-hoc analysis – 03 articles; (12) Non-FOR/NFOR/OTS review – 03 articles; (13) Training management for FOR/NFOR/OTS – 02 articles; (14) Predictive markers of FOR/NFOR/OTS – 02 articles; (15) Pre-stressful events hormonal alterations – 02 articles; (16) Markers of competition performance (but not for FOR/NFOR/OTS) – 01 article; (17) Non-hormonal adaptions to exercise – 01 article; (18) Non-sport FOR/NFOR/OTS – 01 article; (19) In-vitro study – 01 article; and (20) Guideline – 01 article. The total of excluded articles that were not due to duplication were 179 studies, which means among the non-duplicate yielded articles, 82.5% were excluded, whereas 38 (17.5%) were included.

### Study characteristics

#### Sports performed

Among the 38 studies [2–38], six were performed with cyclists, six with triathlon athletes, four with medium distance runners, four with rugby players, three with long distance runners, three with swimmers, three with weight-lifters, three with in military-related physical activities participants, two with soccer players, two with rowing athletes, one in motocross riders, one with short distance runners, one with orienteers, one with skiers, one with tennis players, one with skaters, one with American football players and one with volleyball players (Table 2). Among these studies, six involved more than one modality, whereas two studies did not specify the sports performed in the manuscript. The median number of tested athletes in the 38 studies was 14 (range: 3–36).

**Table 2:** Number of studies performed in each sport

Sport performed	Number of studies	Sport performed	Number of studies
Triathlon	6	Short distance running	1
Cycling	6	Orienting	1
Rugby	4	Motocross	1
Middle distance running	4	Tennis	1
Weight-lifting	3	Skating	1
Long distance running	3	American Football	1
Swimming	3	Karate	1
Multiple military activities	3	Volleyball	1
Soccer	2	More than one sport	6
Rowing	2	Not specified in manuscript	1
Skiing	1		

#### Type of studied athletes and control group

Among the 38 selected studies, 21 (55.2%) were performed in healthy athletes that were induced NFOR/FOR state, while 17 (44.7%) were employed in previously affected athletes; among the 17 articles made with OTS/NFOR/FOR athletes, three (7.9% of total studies) performed an Overload Training Program (OTP) in order to worsen the *underperformance* state, whereas 14 (36.8%) evaluated hormone levels without an OTP.

From the selected studies, 26 (68.4%) had a control group of healthy athletes, whereas 12 (31.6%) compared with previous or basal levels of the same athletes.

#### Type of performed tests

Among the 38 selected studies, 24 (63.2%) evaluated resting hormone levels after an induced NFOR/FOR state, 17 (44.7%) tested basal hormone levels and 16 (42.1%) evaluated acute hormone responses to stimulation tests. Among these, nine (23.7%) evaluated both basal and stimulated hormones, whereas seven (18.4%) tested both acute hormone responses to functional tests and resting levels after an overload training program.

Induction of NFOR/FOR state in healthy athletes was performed either by an OTP (22 studies) or after a Competition Season Period (CSP) (three studies), followed by a proper diagnosis of OTS/NFOR/FOR (reduction of at least 10% of the previous performance). One study [3] performed both OTP and CSP.

Conversely, among the stimulation tests performed in previously affected athletes, 11 (68.8% of all stimulation tests) were carried out with maximal exercise (ME), three (18.8%) with two-bout protocol exercises (TBE) and two (12.5%) with an insulin tolerance test (ITT). Seven of the studies performed two types of stimulation tests.

**Fig. 1:**
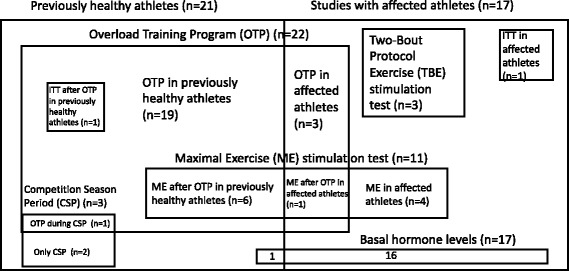
Types of selected athletes and tests performed

Five studies [10, 28, 30, 35, 36] performed both resting levels after an OTP and hormones responses to ME in NFOR/FOR induced athletes by OTP, one [19] performed both basal and stimulated hormones by ITT, and one [18] performed ME response after an OTP, but not basal levels; the seven studies that explored acute responses after an OTP means 33.3% of total of studies that performed OTP; the six studies that performed ME after an OTP represents 54.6% of total studies that employed ME, as the other studies did ME in previously affected athletes without any previous type of overload training. Figure 1 summarizes the types of athletes and tests performed in the selected studies.

Regarding the object of comparison for analysis purposes, 20 studies (52.6%) compared resting hormone levels of FOR-induced athletes by OTP/CSP with previous resting levels; 18 (47.4%) compared basal levels of affected athletes with basal levels of healthy athletes; 12 (31.6%) compared acute hormone responses to stimulation tests between affected and healthy athletes; 11 (28.9%) compared compared resting hormone levels of FOR-induced athletes with resting levels of healthy athletes; and four (10.5%) compared acute hormone response to stimulation tests with basal levels of the same affected athletes. From the 38 studies, 21 (55.2%) performed more than one type of comparison (eg. Analysis between basal levels of the same affected athletes and also between affected and healthy athletes).

### Hormone findings from the selected studies

#### Basal hormones levels

A total of 17 studies (44.7%) compared basal hormone levels between affected and healthy athletes; all hormones and other analyzed parameters disclosed normal levels, as detailed in Table 3.

**Table 3:** Basal hormone levels

Hormone	Quantity of studies	Normal levels	Reduced levels	Increased levels
ACTH	7 (41.2%)	5 (71.4%)	-	2 (28.6%)
Cortisol	6 (35.3%)	4 (66.7%)	1 (16.1%)	1 (16.1%)
GH	5 (29.4%)	3 (60.0%)	1 (20.0%)	1 (20.0%)
NUC	4 (23.5%)	3 (75.0%)	1 (25.0%)	-
IGF-1	2 (11.8%)	2 (100%)	-	-
Total T	2 (11.8%)	1 (50.0%)	1 (50.0%)	-
Plasma Catecholamines	2 (11.8%)	1 (50.0%)	-	1 (50.0%)
Prolactin	2 (11.8%)	2 (100%)	-	-
T/C ratio	2 (11.8%)	2 (100%)	-	-
Insulin	1	1	-	-
SHBG	1	-	-	1
Aldosterone	1	1	-	-
T/SHBG	1	1	-	-
Free T	1	1	-	-
IGFBP-3	1	-	1	-
IGF-1/IGFBP-3	1	-	-	1
Peptide F	1	1	-	-
4 PM salivary C	1	-	-	1

*NUC* Nocturnal urinary catecholamines, *T/C* testosterone/cortisol ratio, *T/SHBG* testosterone/sex hormone binding globulin; *salivary C* salivary cortisol, *4 PM salivary C* salivary cortisol collcted at 4 PM, *IGF-1/IGFBP-3* IGF-1/IGFBP3 ratio

#### Resting hormone levels in NFOR/FOR-induced athletes

A total of 24 studies (63.2%) measured resting hormones of NFOR/FOR-induced athletes and are displayed in Table 4; normal levels were observed in almost all hormones, including the most performed ones: cortisol (75.0% of the findings in 16 studies) and total testosterone (total T) (66.7% of the results in 15 studies), but altered findings were found in testosterone/cortisol ratio (T/C), which has been performed in 10 studies and disclosed reduced ratios in 50.0% of these studies, and in night urinary catecholamines (NUC), which disclosed increased levels in 50.0% of four studies.

**Table 4:** Resting levels after overload training (OTP or CSP)

Parameter	Quantity of studies	Normal response	Blunted response	Exacerbated response
Cortisol	16 (66.7%)	12 (75.0%)	4 (25.0%)	
Total T	15 (62.5%)	10 (66.7%)	4 (26.7%)	1 (6.7%)
T/C	10 (41.7%)	4 (40.0%)	5 (50.0%)	1 (10.0%)
Plasma catecholamines	6 (25.0%)	4 (66.7%)	2 (33.3%)	-
Insulin	5 (20.8%)	4 (80.0%)	1 (20.0%)	-
LH	5 (20.8%)	4 (80.0%)	1 (20.0%)	-
FSH	4 (16.7%)	3 (75.0%)	1 (25.0%)	-
GH	4 (16.7%)	2 (50.0%)	1 (25.0%)	1 (25.0%)
IGF-1	4 (16.7%)	3 (75.0%)	1 (25.0%)	-
NUC	4 (16.7%)	1 (25.0%)	1 (25.0%)	2 (50.0%)
Free T	3 (12.5%)	1 (33.3%)	1 (33.3%)	1 (33.3%)
ACTH	3 (12.5%)	3 (100.0%)	-	-
Aldosterone	3 (12.5%)	1 (33.3%)	1 (33.3%)	1 (33.3%)
T4	2 (8.3%)	2 (100.0%)	**-**	-
T3	2 (8.3%)	2 (100.0%)	**-**	-
TSH	2 (8.3%)	2 (100.0%)	**-**	-
Prolactin	2 (8.3%)	2 (100.0%)	**-**	-
Salivary C	2 (8.3%)	-	2 (100.0%)	-
Salivary T	2 (8.3%)	1 (50.0%)	1 (50.0%)	-
Peptide C	2 (8.3%)	2	-	-
IGFBP-3	1	1	-	-
IGF-1/IGFBP-3	1	1	-	-
DHEA-S	1	1	-	-
Peptide F	1	1	-	-
Fertility	1	-	1	-
SHBG	1	1	-	-
Beta-2 muscle receptors	1	-	1	-

*NUC* Nocturnal urinary catecholamines, *T/C* testosterone/cortisol ratio, *T/SHBG* testosterone/sex hormone binding globulin, *T3* thyronine, *T4* thyroxine, *salivary C* salivary cortisol, *salivary T* salivary testosterone, *4 PM salivary C* salivary cortisol collcted at 4 PM, *IGF-1/IGFBP-3* IGF-1/IGFBP3 ratio, *fertility* evaluation by semen analysis

Finally, T/C and NUC were the only parameters that showed altered levels (reduced T/C and increased NUC) compared to the healthy periods of the athletes.

#### Joint analysis of basal hormone levels and resting hormone levels in NFOR/FOR-induced athletes

For a better comprehension of the findings, studies with basal levels were clustered with studies that performed resting levels of induced NFOR/FOR state in athletes, once both groups present similar diagnosis, regardless of the path (if previously affected or if induced during the study) that led them to the OTS/NFOR/FOR states. Therefore, when analyzed together, basal hormones of previously affected athletes and resting hormones of NFOR/FOR induced subjects displayed normal findings in all parameters, as observed in Table 5.

**Table 5:** Joint of basal with resting hormone levels of athletes induced for NFOR/FOR by overload training

Hormone	Quantity of studies (% of total basal and resting levels)	Normal levels (%)	Reduced levels (%)	Increased levels (%)
Cortisol	22 (53.7%)	16 (72.7%)	5 (22.7%)	1 (4.6%)
Total T	17 (41.5%)	11 (64.7%)	5 (29.4%)	1 (5.9%)
T/C ratio	12 (29.3%)	6 (50.0%)	5 (41.7%)	1 (8.3%)
ACTH	10 (24.4%)	8 (80.0%)	-	2 (20.0%)
GH	9 (21.9%)	5 (55.6%)	2 (22.2%)	2 (22.2%)
NUC	8 (19.5%)	4 (50.0%)	2 (25.0%)	2 (25.0%)
Plasma Catecholamines	8 (19.5%)	5 (62.5%)	2 (25.0%)	1 (12.5%)
IGF-1	6 (14.6%)	5 (83.3%)	1 (16.7%)	-
Insulin	6 (14.6%)	5 (83.3%)	1 (16.7%)	-
LH	5 (12.2%)	4 (80.0%)	1 (20.0%)	-
FSH	4 (9.8%)	3 (75.0%)	1 (25.0%)	-
Free T	4 (9.8%)	2 (100%)	1 (25.0%)	1 (25.0%)
Aldosterone	4 (9.8%)	2 (50.0%)	1 (25.0%)	1 (25.0%)
Prolactin	4 (9.8%)	4 (100%)	-	-
TSH	2 (4.9%)	2 (100%)	-	-
T4	2 (4.9%)	2 (100%)	-	-
T3	2 (4.9%)	2 (100%)	-	-
SHBG	2 (4.9%)	1 (50.0%)	-	1 (50.0%)
IGFBP-3	2 (4.9%)	1 (50.0%)	1 (50.0%)	-
IGF-1/IGFBP-3	2 (4.9%)	1 (50.0%)	-	1 (50.0%)
Peptide F	2 (4.9%)	2 (100%)	-	-
Salivary C	2 (4.9%)	-	2 (100%)	-
Salivary T	2 (4.9%)	1 (50.0%)	1 (50.0%)	-
T/SHBG	1	1	-	-
4 PM salivary C	1	-	-	1
Fertility	1	-	1	-
Beta-2 muscle receptors	1	-	1	-

*NUC* Nocturnal urinary catecholamines, *T/C* testosterone/cortisol ratio, *T/SHBG* testosterone/sex hormone binding globulin, *T3* thyronine, *T4* thyroxine, *salivary C* salivary cortisol, *salivary T* salivary testosterone, *4 PM salivary C* salivary cortisol collcted at 4 PM, *IGF-1/IGFBP-3* IGF-1/IGFBP3 ratio, *fertility* evaluation by semen analysis

#### Acute hormone responses to stimulation tests

Regarding acute stimulated hormonal levels in 16 studies (42.1%), the most employed hormones were cortisol (11 studies), ACTH (seven studies) and 3. GH (seven studies. The detailed findings are shown in Table 6.

**Table 6:** Hormone responses to acute stress (ME, TBE and ITT)

Parameter	Quantity of studies (% of stimulation tests)	Normal response	Blunted response	Exacerbated response
Cortisol	10 (62.5%)	5 (50.0%)	5 (50.0%)	-
ACTH	7 (43.7%)	3 (42.9%)	4 (57.1%)	-
GH	7 (43.7%)	3 (42.9%)	4 (57.1%)	-
Total T	4 (25.0%)	3 (75.0%)	1 (25.0%)	-
Plasma catecholamines	3 (18.7%)	-	2 (66.7%)	1 (33.3%)
Prolactin	3 (18.7%)	1 (33.3%)	2 (67.1%)	-
Insulin	3 (18.7%)	2 (67.1%)	1 (33.3%)	-
LH	3 (18.7%)	3 (100.0%)	-	-
T/C	3 (18.7%)	1 (33.3%)	1 (33.3%)	1 (33.3%)
FSH	2 (12.5%)	2	-	-
IGF-1	2 (12.5%)	1	-	1
LH	2 (12.5%)	2	-	-
FSH	2 (12.5%)	2	-	-
Salivary C	2 (12.5%)	-	2	-
Salivary T	2 (12.5%)	1	1	-
Aldosterone	2 (12.5%)	-	1	1
IGFBP-3	1	-	1	-
IGF-1/IGFBP-3	1	-	-	1
Fertility	1	-	1	-

*NUC* Nocturnal urinary catecholamines, *T/C* testosterone/cortisol ratio, *T/SHBG* testosterone/sex hormone binding globulin, *T3* thyronine, *T4* thyroxine, *salivary C* salivary cortisol, *salivary T* salivary testosterone, *IGF-1/IGFBP-3* IGF-1/IGFBP3 ratio, *fertility* evaluation by semen analysis

Herein, GH, ACTH and prolactin shows undoubtedly blunted responses to most acute stressful tests, despite of the small number of studies and subjects evaluated, whereas other hormones show normal findings.

#### Joint analysis of resting hormone levels in NFOR/FOR-induced athletes and acute hormone responses to stimulation tests

Acute hormone responses to stimulation tests can also be analyzed together with resting hormone levels after an OTP, once both explore capacity to respond to stressful situations. When acute hormones responses were analyzed together with the resting levels of induced NFOR/FOR, the only marker that showed mostly altered levels was aldosterone. However, contradictory findings were observed since half of the altered results were increased whereas half were decreased. Detailed findings are displayed in Table 7.

**Table 7:** Joint of acute hormone responses to stimulation tests and resting levels after induction of NFOR/FOR state

Hormone	Quantity of studies	Normal response	Blunted response	Exacerbated response
Cortisol	26 (65.0%)	18 (69.2%)	8 (30.8%)	-
Total T	18 (45.0%)	14 (77.8%)	3 (16.7%)	1 (5.9%)
GH	13 (32.5%)	7 (53.8%)	5 (38.4%)	1 (7.8%)
T/C	10 (25.0%)	5 (50.0%)	4 (40.0%)	1 (10.0%)
ACTH	10 (25.0%)	6 (60.0%)	4 (40.0%)	-
Plasma catecholamines	9 (22.5%)	4 (44.4%)	4 (44.4%)	1 (11.1%)
Insulin	8 (20.0%)	6 (75.0%)	2 (25.0%)	-
LH	8 (20.0%)	7 (87.5%)	1 (12.5%)	-
Prolactin	6 (15.0%)	4 (66.7%)	2 (33.3%)	-
FSH	6 (15.0%)	5 (83.3%)	1 (16.7%)	-
IGF-1	5 (12.5%)	3 (60.0%)	1 (20.0%)	1 (20.0%)
Aldosterone	5 (12.5%)	1 (20.0%)	2 (40.0%)	2 (40.0%)
Free T	3 (7.5%)	1 (33.3%)	1 (33.3%)	1 (33.3%)
T4	2 (5.0%)	2 (100%)	-	-
T3	2 (5.0%)	2 (100%)	-	-
NUC	2 (5.0%)	-	2 (100%)	-
Salivary C	2 (5.0%)	-	2 (100%)	-
Salivary T	2 (5.0%)	1 (50.0%)	1 (50.0%)	-
NUC	2 (5.0%)	1 (50.0%)	1 (50.0%)	-
IGFBP-3	2 (5.0%)	1 (50.0%)	1 (50.0%)	-
IGF-1/IGFBP-3	2 (5.0%)	2 (100%)	-	-
SHBG	1	1	-	-
DHEA-S	1	1	-	-
Peptide F	1	1	-	-
Fertility	1	-	1	-
Beta-2 muscle receptors	1	-	1	-

*NUC* Nocturnal urinary catecholamines, *T/C* testosterone/cortisol ratio, *T/SHBG* testosterone/sex hormone binding globulin, *T3* thyronine, *T4* thyroxine, *salivary C* salivary cortisol, *salivary T* salivary testosterone, *IGF-1/IGFBP-3* IGF-1/IGFBP3 ratio, *fertility* evaluation by semen analysis

#### Hormonal levels of the type of athletes (if previously healthy or affected by OTS/NFOR/FOR)

Whether athletes were previously affected by OTS/NFOR/FOR or if they were previously healthy and were induced to NFOR/FOR states by an OTP or CSP may be meaningful to the hormone findings. Table 8 shows the hormonal results according to the type of selected athletes, regardless of being basal, resting or acutely stimulated, once the objective in this section is to evaluate hormone levels as a whole in previously affected compared to previously healthy athletes. For analysis purposes, we considered only hormones that have been performed by more than one study While 14 out of 16 hormones (87.5%) were mostly normal in FOR-induced athletes, five from 12 hormones (41.7%) disclosed mostly normal levels and responses when OTS-affected athletes were analyzed.

**Table 8:** Results according to tests performed and subjects analyzed

Parameter	FOR-induced athletes	Previously affected subjects
Cortisol	Normal results – 11 (68.8%) Reduced levels – 5 (31.2%) Total - 16	Normal results – 7 (50%) Increased levels – 2 (14.3%) Reduced levels – 5 (35.7%) Total - 14
ACTH	Normal results – 3 (100%)	Normal results – 6 (46.2%) Increased levels – 2 (15.3%) Reduced levels – 5 (38.5%) Total – 13
Serum catecholamines	Normal results – 4 (66.7%) Reduced levels – 2 (33.3%) Total - 6	Normal results – 1 (20.0%) Increased levels – 2 (40.0%) Reduced levels – 2 (40.0%) Total – 5
GH	Normal results – 5 (100%) Total - 5	Normal results – 1 (12.5%) Increased levels – 2 (25.0%) Reduced levels – 5 (62.5%) Total - 8
T/c	Normal results – 5 (62.5%) Reduced levels – 3 (37.5%) Total - 8	Normal results – 1 (33.3%) Increased levels – 1 (33.3%) Reduced levels – 1 (33.3%) Total - 3
SHBG	-	Increased levels – 1 (100%)
IGF-1	Normal results – 1 (50.0%) Reduced levels – 1 (50.0%) Total - 2	Normal results – 1 (33.3%) Increased levels – 2 (66.7%) Total - 3
Free T	Normal results – 1 (50.0%) Increased levels – 1 (50.0%) Total - 2	Reduced levels – 1 (100%)
Salivary C	-	Reduced levels – 2 (100%)
Salivary T	-	Increased levels – 1 (50.0%) Reduced levels – 1 (50.0%)
Total T	Normal results – 11 (78.6%) Increased levels – 1 (7.1%) Reduced levels – 2 (14.3%) Total - 14	Normal results – 1 (33.3%) Reduced levels – 2 (66.7%) Total - 3
Insulin	Normal results – 6 (85.7%) Reduced levels – 1 (14.3%) Total - 7	Normal results – 4 (80.0%) Reduced levels – 1 (20.0%) Total - 5
NUC	Increased levels – 1 (25.0%) Reduced levels – 3 (75.0%) Total - 4	Normal results – 2 (100%)
IGFBP-3	-	Reduced levels – 1 (100%)
IGF-1/IGFBP-3	-	Increased levels – 1 (100%)
Prolactin	Normal results – 4 (66.7%) Reduced levels – 2 (33.3%) Total - 6	Normal results – 1 (50.0%) Reduced levels – 1 (50.0%) Total - 2
LH	Normal results – 6 (85.7%) Reduced levels – 1 (14.3%) Total – 7	-
FSH	Normal results – 4 (80.0%) Reduced levels – 1 (20.0%) Total - 5	-
Aldosterone	Normal results – 1 (16.7%) Increased levels – 2 (33.3%) Reduced levels – 3 (50.0%) Total - 6	-
DHEA-S	Normal results – 1 (100%)	-
Peptide F	-	Normal results – 1 (100%)
Fertility	Reduced levels – 1 (100%)	-
TSH	Normal results – 4 (100%)	-
T4 L	Normal results – 2 (100%)	-
T3 L	Normal results – 2 (100%)	-
16 h salivary C	Increased levels – 1 (100%)	-
Beta2 muscle receptor	Reduced levels – 1 (100%)	-
Total number of studies	21 (55.3%)	17 (44.7%)

## Discussion

### Systematic search

The aim of this systematic review is to evaluate hormonal aspects of OTS/NFOR/FOR already published. Therefore, after the search for a wide number of expressions and hormones, only 38 met the criteria, as many expressions yielded the same studies. Moreover, 82.5% of the studies were descriptive, did not perform hormone tests, or therefore did not add new information or evidence to correlation between OTS/NFOR/FOR and hormone profile. The systematic search was expanded beyond the PRISMA guidelines for systematic reviews in order to achieve all the studies that correlate hormone levels and OTS/NFOR/FOR, due to the wide variety of expressions and study designs.

As stated in the results section, it was unfeasible to analyze each sport separately due to the small number of subjects of each study, wide distribution of sports performed, heterogeneity regarding most aspects of the studies, such as inclusion criteria, and precise definition of who were affected by OTS/NFOR/FOR.

Interestingly, more reviews on hormonal aspects of OTS/NFOR/FOR were found (55 reviews) than the number of selected studies (44.7% more than original articles). Moreover, five studies improperly diagnosed OTS/NFOR/FOR based on hormones alterations, such as reductions of 30% or more of T/C ratio, regardless of the performance status.

### Types of parameters and tests performed

There were three types of assessed hormones: 1. basal hormones in previously affected athletes; 2. resting levels after an overload training; and 3. acute hormone responses to stimulation tests in OTS/NFOR/FOR individuals.

Among the 38 selected studies, 26 different hormones and hormone-related parameters were assessed, both basally and stimulated. Interestingly, the stimulation tests performed were not those recommended by endocrine societies and have not been validated previously, except two, that performed ITT; indeed, the oldest study found by this systematic review, performed by Barron et al. [38] and published in 1985 performed ITT and found blunted ACTH and cortisol levels in OTS/NFOR athletes; however, no further studies that tried to reproduce these findings were found. ITT is a gold-standard functional test recommended by Endocrine societies to evaluate stress hormone response to a simulation of a stressful situation, the hypoglycemia. This test requires the whole axis integrity in order to provide normal responses; and, regardless of the affected level of each axis, the lack of response mimic the real-life stressful situations, which may lead to decreased performance in extreme sports sessions, when integrity of stress-related axis are required.

Although not supported by Endocrine societies, some authors showed that hormonal responses to intense exercises (ME) seemed to be more appropriate to evaluating OTS/NFOR/FOR, which is confirmed by the findings of this systematic review. Thus five different kinds of stimulation tests were used for the evaluation of hormonal responsiveness, including three that evaluated acute response tests (ME, TBE and ITT), and two that measured in resting after a training overload (OTP and CSP). According to the most recent OTS/NFOR/FOR guidelines [1], the most cited test among the reviewed studies was TBE; however, we observed that ME, and not TBE, was the most studied functional test, and was able to show significant differences between OTS/NFOR/FOR and healthy athletes. However, none of the studies established a proposed cutoff for any of the tested hormones, and most of the conclusions recommend further studies to establish validated cutoff and criteria.

The heterogeneity of ME protocols, the lack of cutoff for the tests hormones (despite of the altered hormone findings), and the small number of participants in most studies (as it is not easy to recruit athletes affected with OTS/NFOR/FOR in the current moment of the disease) lead to the fact that is is possibl but uncertain whether the conducted tests are useful for the accurate diagnosis of OTS/NFOR/FOR. Further studies should explore the findings of this systematic review and perform validated tests by Endocrine societies, such as ITT, and standardized tests by the latest guideline [1], such as TBE, and provide correlation between responses of thes two types of tests, as well as attempt to establish reliable cutoffs for each hormone in order to provide a new biomarker for OTS/NFOR/FOR.

### Types of sports practiced

OTS/NFOR/FOR related to endurance sports was analyzed by 35 (92.1%) studies, whereas resistive exercises were only analyzed by three (7.9%), accordingly with the lack of studies described by the latest guidelines [1].

Regarding endurance exercises, despite the potentially high prevalence of OTS/NFOR/FOR among triathlon athletes, only six studies performed tests in this population, whereas other sports which OTS/NFOR/FOR has been less described have been perhaps disproportionately studies.

Unlike in endurance sports, that showed reduction in catecholamines levels, an increase was observed in weight lifters affected by OTS/NFOR, as seen in the three studies that were performed in resistive athletes (weight-lifters), which distinct results could be observed from the other studies, once the two studies that evaluated catecholamines found increased levels, and decreased beta-receptor concentrations (*down-regulation*) in muscles were found in one, with consequent decreased sensitivity to catecholamines. This finding is corroborated by Fry et al. [17], whose correlation coefficients suggested decreased responsivity of skeletal muscle to sympathetic nervous system activity and therefore corroborates the sympathetic aspect of the resistive FOR. Therefore, the named sympathetic OTS/NFOR/FOR for resistive athletes [1, 39] may be appropriate, although further studies are necessary to corroborate these findings. as there is a lack of previous controlled studies with resistive exercises.

### Hormones and OTS/NFOR/FOR

#### Establishment of cutoffs

Cutoffs developed for hormone levels as OTS/NFOR/FOR markers would thus be distinct from the cutoffs for the normal ranges used to diagnose endocrine conditions (1,4,7,10,11), as frank low or high hormone levels lead to diagnosis of endocrine dysfunction, which initially exclude OTS/NFOR/FOR, and symptoms could be possibly explained by the underlying disease [1, 39]. Therefore, regardless of the normal range, whenever athletes with OTS/NFOR/FOR presented significantly different hormone levels than healthy athletes, basal hormones would be able to be good markers or predictors of OTS/NFOR/FOR, and therefore cutoffs could be established using specific statistics mechanisms.

#### Basal levels

Among all studies, basal levels of 17 tested hormones (Table 6) were normal between affected and healthy athletes in at least 75.0% of the studies for each hormone, with the exceptions of plasma catecholamines, which was performed in only two studies and found increased levels in one (50.0%), and one study that employed IGFBP-3 and found reduced levels. Therefore, none of the evaluated basal hormone levels, nor the hormone-related parameters, appear to be good predictors of OT/OR.

#### Resting levels in FOR-induced athletes

Resting levels performed in NFOR/FOR-induced athletes are hormones that are measured after a night sleep in the same conditions as the basal hormones, in subjects that are induced for NFOR/FOR states after an overload training period, as part of the study intervention, and were demonstrated to show decrement in performance capacity. Herein, 24 studies employed this method, and normal levels were seen in all parameters, except for T/C ratio, which was successful to show altered ratios compared to healthy athletes in 50.0% of the studies (40.0% showed reduced ratios and 10.0% showed increased ratios), while normal findings were observed in 50.0%.

When analyzed together, basal and resting hormones were mostly normal, once none of the parameters were shown to be altered in more than 50.0% of the selected articles, whenever three or more studies were found for the analyzed marker.

#### Stimulated hormone responses as markers of OTS/NFOR/FOR

In contrast to basal levels, blunted hormone acute responses were seen in OTS/NFOR/FOR athletes, notably in prolactin (67.1% of the studies), in GH (57.1%) and in ACTH (57.1%) levels, whereas conflicting results in plasma catecholamines (50.0% of responses were blunted, 25.0% were increased and 25.0% normal) and in cortisol responses (54.6% of tests were normal and 45.4% were blunted), in comparison to healthy subjects. Notably, acute blunted responses were observed regardless of the type of tests performed, although most employed tests were not validated or supported by endocrine societies. Therefore, further studies are necessary to establish reliable markers and specific cutoffs for each potentially accurate marker.

### The role of hormones in overtraining and overreaching syndromes

The lack of alterations in basal and resting levels in OTS/NFOR/FOR athletes may be explained by the high adaptive capacity of athletes to extreme environments, which leads to shorter periods of recovery after physical exertion. The basal hormone release may not be altered in these subjects, distinctly from the possible impaired capacity to respond to stressful situations.

Indeed, despite of the limitations found in this systematic review, the consistent findings of blunted ACTH, GH and prolactin levels observed in all stimulation tests deserve attention. Hormones and glands physiology present similarities among them in terms of basal and stimulated hormone production and release. In this case, initial stages of glands dysfunctions tend to preserve basal hormone production, but present impaired responses to stimulations, such as stressful situations. Hormonal alterations may be therefore only seen in acute responses to functional tests, but not in basal and in resting levels, as observed in relative adrenal insufficiency, GH deficiency and pre-diabetes and initial diabetes. Relative adrenal insufficiency shows as a lack of enough raise in cortisol levels to ITT or to ACTH-stimulation test; GH deficiency is diagnosed when a blunted GH is observed to ITT; and pre-diabetes or initial diabetes are more accurately diagnosed by Oral Glucose Tolerance Test, which provides earlier information about disglycemia than fasting serum glucose, once relative hypoinsulinemia with consequent hyperglycemia in response to oral glucose is observed. In fact, severe adrenal insufficiency and final stage of diabetes are required to present basal hypocortisolism and fasting hyperglycemia with inappropriate hypoinsulinemia, respectively. TRH stimulation test for thyroid function and LHRH stimulation test for ovary function are able to show depletion of these glands prior to basal levels as well. Therefore, it is possible, if not probable, that compromised glands with relative hormone dysfunction are present in OTS, although further studies are required, particularly linking acute hormonal responses to exercises with functional tests standardized by endocrine societies.

Notably, hormonal changes in OTS/NFOR/FOR are probably not triggers of these disorders, but may play a role in the worsening and perpetuation of OTS/NFOR/FOR symptoms. Thus once identified as markers, hormones should not be replaced; instead, their recovered levels could be used as markers of improvement in OTS/NFOR/FOR. Additionally, once basal levels are altered, a diagnosis of OTS/NFOR/FOR is unlikely, as these conditions should not be diagnosed in the presence of endocrine alterations [1, 4, 28].

Although acute responses to stress in OTS/NFOR/FOR athletes and resting levels responses to NFOR/FOR state were also analyzed together, they may present distinct results, once they were measured in different situations, allied to the fact that OTS may differ biochemically from NFOR/FOR states. Indeed, one study showed different results between OTS and NFOR/FOR [9], once in OTS, GH and ACTH failed to raise in response to a TBE, whereas in NFOR/FOR athletes both hormones markedly raised in response to TBE. It was also observed that studies with previously affected athletes tend to show more pronounced altered responses than studies that induces FOR/NFOR, perhaps due to the above-mentioned possible difference between FOR (when overload is performed, a FOR/NFOR state is induced, not OTS) and OTS (most studies with previously affected subjects tended to include OTS, not FOR/NFOR athletes).

### Differential diagnosis

Many dysfunctions that may confound the proper OTS/NFOR/FOR diagnoses have to be excluded in athletes that also lead to worsened performance and fatigue, including: 1. Sleep disorders; 2. Site-specific or sub-clinical diffuse inflammation (non-alcoholic steatohepatitis – NASH, metabolic syndrome, dyslipidemia); 3. Frank hormonal dysfunctions; 4. Autoimmune diseases (rheumatoid arthritis, systemic lupus erythematosus, ankylosing spondylitis); 5. Vitamins deficiency (vitamin B12, B1, D); 6. Psychiatric conditions (bipolar disorder, depression, anxiety disorders); 7. Lung dysfunctions (asthma, idiopathic pulmonary fibrosis, tuberculosis) and 8. Heart conditions (persistence space of double interatrial septum, heart failure) Most of the above listed differential diagnosis are not explicitly exposed in the latest OTS/NFOR/FOR guideline [1].

Fatigue is not always linked to reduced performance, and hormonal differences may be observed between non-reduced performance and reduced-performance fatigued athletes. Aubry et al. [2] compared athletes acutely fatigued without decrement of performance and FOR athletes, and catecholamines were found to be reduced in FOR compared to the non-FOR fatigued subjects.

### Hormonal markers of OTS/NFOR/FOR: Specific for these conditions?

Some alterations found in OTS/NFOR/FOR athletes may be resulted from overload training, regardless of the performance state, and therefore be also observed in healthy athletes. Indeed, Uusital et al. [28] performed an Experimental Training Group (ETG) with massively intense exercises (above lactate threshold) compared to control group. Cortisol and noradrenaline were decreased in the ETG in both induced FOR (*n* = 5) and non-FOR (*n* = 4), compared to control, which means that altered findings may not always differentiate OTS/NFOR/FOR, but indicate an excess of training. The proposed markers of OTS/NFOR/FOR were also contested by Hoogeveen et al. [30], who showed that hormonal changes were the same between OTS and healthy athletes, concluding that all the alterations were related to physiological adaptions, not to reduced sports performance.

Furthermore, despite of the systematic search for decreased total T and T/C ratio as markers of worsened performance, Hoogeveen et al. [30] showed that decreased total T, increased cortisol and decreased T/C ratio failed to predict reduction in performance.

### Limitations

Some remarkable limitations in studies that evaluated hormonal levels in OTS/NFOR/FOR were: 1) The impossibility of performing a meta-analyses owing to the wide variety of methodologies; 2) Wide variety of evaluated sports; 3) Lack of standardization criteria to differentiate between OTS, NFOR and FOR; 4) Lack of enough studies comparing OTS/NFOR/FOR with healthy athletes and healthy sedentary individuals, in order to evaluate physiological hormone adaption via training; 5) Small numbers of participants in most studies; 6) Lack of studies with sympathetic OTS/NFOR/FOR (such as in weight-lifters); and 7) Lack of standardized stimulation tests endorsed by endocrinology societies, to enforce the evidence strength.

Besides the limitations of the selected studies, this systematic review also presented some limitations. First, if the basic PRISMA protocol of search for systematic reviews was followed, only 12 studies would have been selected. Therefore, an expanded search that went beyond an usual systematic review had to be performed. Second, a more comprehensive analysis for more precise conclusions was unable to be performed, due to the unexpected differences in methodology, even when types of sports or tests were similar. And third, we found less consistent data then expected, which did not allow a more complete systematic review in the field.

### Final discussion

To date, this is the first systematic review that evaluated hormonal aspects of OTS./NFOR/FOR. The large number of studies that compared basal levels between affected and healthy athletes may lead to the conclusion that basal levels of hormones are definitely not good markers of OTS/NFOR/FOR. Conversely, stimulated hormones, particularly acute responses to stressful conditions may be good predictors of underperformance syndromes, although OTS/NFOR/FOR is a complex disorder, with multi-etiologic pathophysiology, and therefore hormones dysfunctions are unlikely to be the only etiology of OTS/NFOR/FOR.

The overall conflicting findings of the present systematic review reflects the complex relation between hormones and overtraining syndrome. The main issue regarding hormone findings in OTS is the causality relationship and the real underlying reason that leads to decreased performance. Whenever the hormone dysfunction is likely the primary cause for the worsened performance, OTS is excluded, once OTS requires exclusion of endocrine disorders to be diagnosed. Conversely, OTS may lead to dysfunctional hormones, as observed [1–38], and identifying whether these dysfunctions are secondary to OTS is challenging. As endocrinologists, we stated that generally, whenever a frank and severe hormone dysfuncion was found, the diagnosis of OTS is unlikely, once OTS tend to induce mild changes in hormone, not important changes, and since the hormone dysfuncion probably explains all the clinical findings, and organic etiologies for the dysfuncion should be searched. Finally, who defines the diagnosis is the underlying etiology: whenever the hormone dysfuncions do not fully explain the clinical presentation, and seem to be a consequence of the OTS, OTS is the diagnosis; contrariwise, if the clinical presentation is attributable to the hormone dysfunctions, the replacement or suppression of the affected hormone should be approached prior to the diagnosis of OTS.

Finally, it may be too early to claim that GH, ACTH and prolactin responses are undoubtedly blunted in acute responses in affected individuals, once not all studies found the same results, studies performed slightly different protocols for ME and TBE, and small number of subjects were included. For practical purposes, whenever an athlete is suspected for OTS/NFOR/FOR, stimulation tests could be performed in order to find possible relative failure of the adrenals or the pituitary, although only standardized tests by Endocrine societies (ITT) are enough to provide these diagnosis.

Further studies should be performed with athletes from the sports that are mostly described in OTS/NFOR/FOR, such as triathlon, cycling and long distance runners, standardization of ME should be employed, both ME and ITT should be performed and correlated, and a specific control group of healthy athletes that practice the equivalent intensity and volume of training should also perform the exact same tests, to strength the level of the found data.

## Conclusion

Acute hormone responses to stimulation tests, such as ACTH, GH and prolactin, tend to be blunted in OTS/NFOR/FOR, whereas cortisol and plasma catecholamines presented conflicting results, and other hormonal acute responses were not systematically analyzed. The most performed stimulation test was ME, differently from what the latest guidelines on OTS/NFOR/FOR stated. The dysfunctional responses observed in different sorts of stimulation tests may demonstrate a relative failure of hormonal axis. Contrariwise, basal and resting parameters do not seem to play an accurate tool for OTS/NFOR/FOR diagnosis.

Despite of these conclusions, further studies are needed in order to improve hormonal markers accuracy and cutoffs. This systematic review will help future studies to draw more specific targets and accurate methods.

## Abbreviations

CB: Compared to Basal levels of the same subjects, when previously not affected
CC: Compared to healthy Control (both basal and estimulated)
CSP: Competition Season Period
FOR: Functional overreaching
I: Increased
iFOR: induced NFOR/FOR states
IPT: Immediately Post Training
ITT: Insulin tolerance test
ME: Maximal Exercise
N: Normal
N: Number of subjects
NFOR: Non-functional overreaching
Nl: Normal levels
NS: Not specified
NUC: Nocturnal urinary catecholamines
OTP: Overload Training Program
OTS: Overtraining syndrome
pAFFECT iFOR: Induced NFOR/FOR in previously affected subjects
pAFFECT: Previously Affected Subjects
R: Reduced
T/C: Testosterone/cortisol
T/SHBG: Testosterone/sex hormone binding globulin
TBE: Two-bout protocol exercise
